# P-2023. A ‘Variant-Proof’ Cocktail Of Two Heavy Chain-Only Antibodies Targets 3 Epitopes In The Spike Protein S1 And S2 Subunits And Broadly Neutralizes SARS-Cov-2 With Exceptional Potency

**DOI:** 10.1093/ofid/ofae631.2179

**Published:** 2025-01-29

**Authors:** Toon Venneman, Florence M Herschke, Rinaldi Manuela, Van Royen Tessa, Christophe Blanchetot, Bert Schepens, Sieglinde De Cae, Kenny Roose, Loes van Schie, Dirk M Reiter, Inge Van Molle, Han Remaut, Nico Callewaert, Xavier Saelens, Viki Bockstal

**Affiliations:** ExeVir, Gent, Oost-Vlaanderen, Belgium; ExeVir Bio, Gent, Oost-Vlaanderen, Belgium; Exevir, Zwijnaarde, Oost-Vlaanderen, Belgium; ExeVir, Gent, Oost-Vlaanderen, Belgium; exevir, Gent, Oost-Vlaanderen, Belgium; VIB/Ghent University, Ghent, Oost-Vlaanderen, Belgium; VIB-UGent, Gent, Oost-Vlaanderen, Belgium; VIB-UGent, Gent, Oost-Vlaanderen, Belgium; VIB-UGent, Gent, Oost-Vlaanderen, Belgium; VIB&VUB, Brussels, Brussels Hoofdstedelijk Gewest, Belgium; VUB, Brussels, Brussels Hoofdstedelijk Gewest, Belgium; VIB-VUB, Brussels, Brussels Hoofdstedelijk Gewest, Belgium; VIB-UGent, Gent, Oost-Vlaanderen, Belgium; VIB-UGent, Gent, Oost-Vlaanderen, Belgium; ExeVir, Gent, Oost-Vlaanderen, Belgium

## Abstract

**Background:**

More than 7 million people have died from COVID-19 to date and SARS-CoV-2 continues to cause substantial disease around the world. High-risk patients like immunocompromised and elderly are often not able to elicit adequate immune responses to vaccination and strongly benefit from an additional layer of protection in the form of complementary antibody therapy. Monoclonal antibodies act immediately and can provide immune support for months. However, all previously authorized antibodies lost their neutralization potency against the currently circulating and constantly mutating SARS-CoV-2 variants, leaving a vast unmet medical need.

The combination XVR012 consists of the molecules XVR013m and XVR014.

**Figure:**
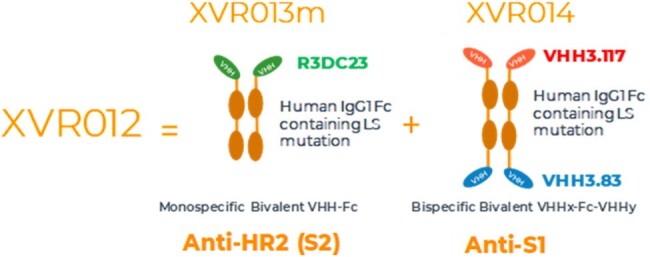

**Methods:**

The combination XVR012 consists of the molecules XVR013m and XVR014 (Figure 1). XVR013m is a mono-specific VHH-Fc antibody that targets a unique epitope in the S2 subunit, XVR014 is a bi-specific bivalent VHHx-Fc-VHHy antibody construct, targeting 2 non-competing epitopes in the Receptor-Binding Domain of the spike (Figure 2). Both antibodies contain a human IgG1 Fc with LS mutations for half-life extension.

Structural analysis of the XVR013m epitope

**Figure:**
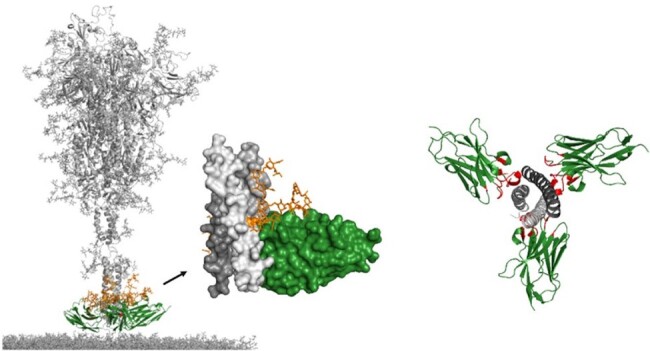

Left panel: Structural analysis of the VHH R3DC23 (green) bound to the HR2 domain of the S2 unit of the SARS-CoV-2 spike protein, between the terminal N1194 glycan (orange) and the viral membrane. Right panel: One R3DC23 (green) binds the interface of two HR2 coils and each HR2 coil is bound by two R3DC23 VHHs.

**Results:**

XVR012 delivers a triple mode of action in preventing infection of host cells, i.e., (1) sterical hinderance of ACE2 receptor binding, (2) induction of S1 subunit shedding thereby preventing viral attachment and (3) inhibition of the fusion process between the viral and host cell membrane. XVR012 broadly neutralizes SARS-CoV-1 and SARS-CoV-2 viruses *in vitro* and demonstrates an IC50 ranging from 4.8 to 8,7 ng/mL against all SARS-CoV-2 variants tested so far in a pseudovirus neutralization assay, including the currently circulating BA.2.86.1, HK.3, EG.5.1 and HV.1 (Table 1). *In vivo*, efficacy was demonstrated in the Syrian Golden Hamster model in a therapeutic setting, showing complete reduction of lung viremia for all XVR012 dose levels tested, even with a very low dose of 0,5 mg/kg for the XVR013m component. (Figure 3). PK parameters have been determined in a Tg32 SCID mouse model and support an envisioned duration of protection up to 6 months.

Neutralization potency of VSV pseudo-typed with spike proteins of recent SARS-CoV-2 variants.

**Figure:**
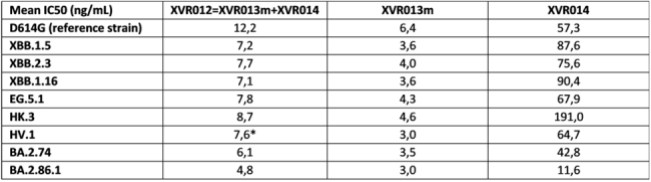

Data are presented as mean IC50 based on 3 independent experiments, except for (*) 1 independent experiment.

**Conclusion:**

In summary, this second-generation cocktail targeting three unique and highly conserved epitopes in the S1 and S2 subunits of the spike is ready to proceed to clinical testing and may provide a long-term solution to the populations at highest risk.

Lung infectious viral titers in Syrian golden hamster post-infection challenge model (Wuhan strain) on day 4 post infection.

**Figure:**
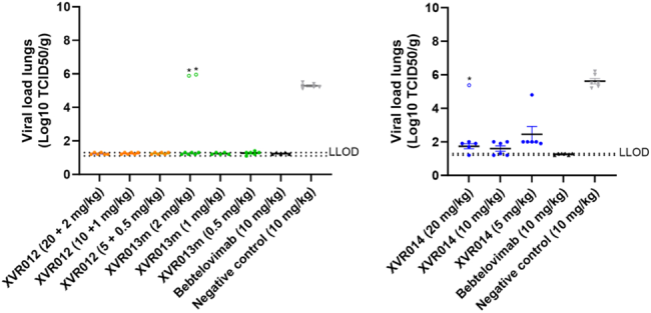

Mean values + standard error of the mean (SEM) are reported. * Two animals in 2 mg/kg group of XVR013m and one animal in the 20 mg/kg group of XVR014 were experimentally confirmed in PK assays to not have been exposed to the drug. Dotted lines represent the lower limit of detection (LLOD) range.

**Disclosures:**

Florence M. Herschke, PhD, ExeVir Bio: Patent inventor|ExeVir Bio: Employee Bert Schepens, PhD, Exevir: Bert Schepens is Inventor on patents that describe the nanobodies that are mentioned in the abstract and presentation Loes van Schie, PhD, Exevir: Grant/Research Support|Exevir: I am a co-inventor on the patents protecting the biologicals described in the abstract and presentation Xavier Saelens, PhD, ExeVir Bio: I am an inventor on patent applications WO2022/167666 A1 and WO2023/222825 A1 which incorporate discoveries and inventions described here.|ExeVir Bio: I am a scientific co-founder of ExeVir Bio and in receipt of ExeVir Bio share options

